# REST/NRSF Knockdown Alters Survival, Lineage Differentiation and Signaling in Human Embryonic Stem Cells

**DOI:** 10.1371/journal.pone.0145280

**Published:** 2015-12-21

**Authors:** Kaushali Thakore-Shah, Tasneem Koleilat, Majib Jan, Alan John, April D. Pyle

**Affiliations:** 1 Molecular Biology Institute, University of California Los Angeles, Los Angeles, CA, 90095, United States of America; 2 California State University, Northridge, CA, 91325, United States of America; 3 Department of Microbiology, Immunology, and Molecular Genetics, University of California Los Angeles, Los Angeles, CA, 90095, United States of America; 4 Eli and Edythe Broad Center of Regenerative Medicine and Stem Cell Research, Jonsson Comprehensive Cancer Center, University of California Los Angeles, Los Angeles, CA, 90095, United States of America; University of Illinois at Chicago, UNITED STATES

## Abstract

REST (RE1 silencing transcription factor), also known as NRSF (neuron-restrictive silencer factor), is a well-known transcriptional repressor of neural genes in non-neural tissues and stem cells. Dysregulation of REST activity is thought to play a role in diverse diseases including epilepsy, cancer, Down’s syndrome and Huntington’s disease. The role of REST/NRSF in control of human embryonic stem cell (hESC) fate has never been examined. To evaluate the role of REST in hESCs we developed an inducible REST knockdown system and examined both growth and differentiation over short and long term culture. Interestingly, we have found that altering REST levels in multiple hESC lines does not result in loss of self-renewal but instead leads to increased survival. During differentiation, REST knockdown resulted in increased MAPK/ERK and WNT signaling and increased expression of mesendoderm differentiation markers. Therefore we have uncovered a new role for REST in regulation of growth and early differentiation decisions in human embryonic stem cells.

## Introduction

REST (RE1 silencing transcription factor), also known as NRSF (neuron-restrictive silencer factor), is a zinc-finger transcription factor that regulates expression of a diverse set of genes in a tissue specific manner [1–4]. It binds to a 21–23 base pair DNA motif, known as repressor element 1 (RE1; also known as NRSE) [5,6], of which there are at least 1900 copies in the human genome [1,7,8]. REST was originally identified as a transcriptional repressor of neural genes in non-neural tissues and stem cells [9–11]. It has since been demonstrated to be aberrantly expressed in various cancers, and is now recognized to play a tumor suppressor role in epithelial cells, and an oncogenic role in neural cells [2, 12–15]. In addition, dysregulation of REST and its cofactors is implicated in the molecular pathophysiology of various diseases such as cardiac hypertrophy [16], ischemia [17], epilepsy [18, 19], Down’s syndrome [20], Huntington’s disease [21, 22], and X-linked mental retardation [23].

In mouse embryonic stem cells (mESCs), modulation of REST protein levels can regulate the transition from a pluripotent stem cell to a neural progenitor cell and from progenitor to mature neuron [9]. Loss of Rest using conventional Rest knockout mice leads to the early embryonic lethality [11]. Using conditional knockout mice, it has been shown that Rest plays a role in suppressing the expression of neuronal genes in cultured neuronal cells *in vitro*, as well as in non-neuronal cells outside of the central nervous system, but that it is dispensable for embryonic neurogenesis *in vivo* [24]. Studies in mESCs showing that Rest is directly regulated by the core pluripotency transcription factors Oct4, Sox2, and Nanog [25], that Nanog is a direct Rest target, and that 107 genes including Rest itself are targets of all four factors [26], provide strong evidence that REST is an integral part of the stem cell regulatory network.

In one study, REST has been shown to play a role in maintaining self-renewal and pluripotency of mESCs, partly by repressing neuronal differentiation [27]. However, other groups have found REST to mainly regulate lineage specification from mESCs [28], and the role of REST in pluripotency has been the topic of much debate [29]. In human embryonic stem cells (hESCs), the core transcriptional regulators OCT4, SOX2 and NANOG, have been shown to bind to the REST promoter [30]. Based on the association with the pluripotency network, we expected that REST would be important for maintaining pluripotency in hESCs. Our data suggest that REST is not essential for maintenance of self-renewing stem cells but that REST levels are important for regulation of survival. We have also uncovered a new role for REST in regulation of the early events of lineage differentiation and signaling in hESCs.

## Results

**Fig 1:**
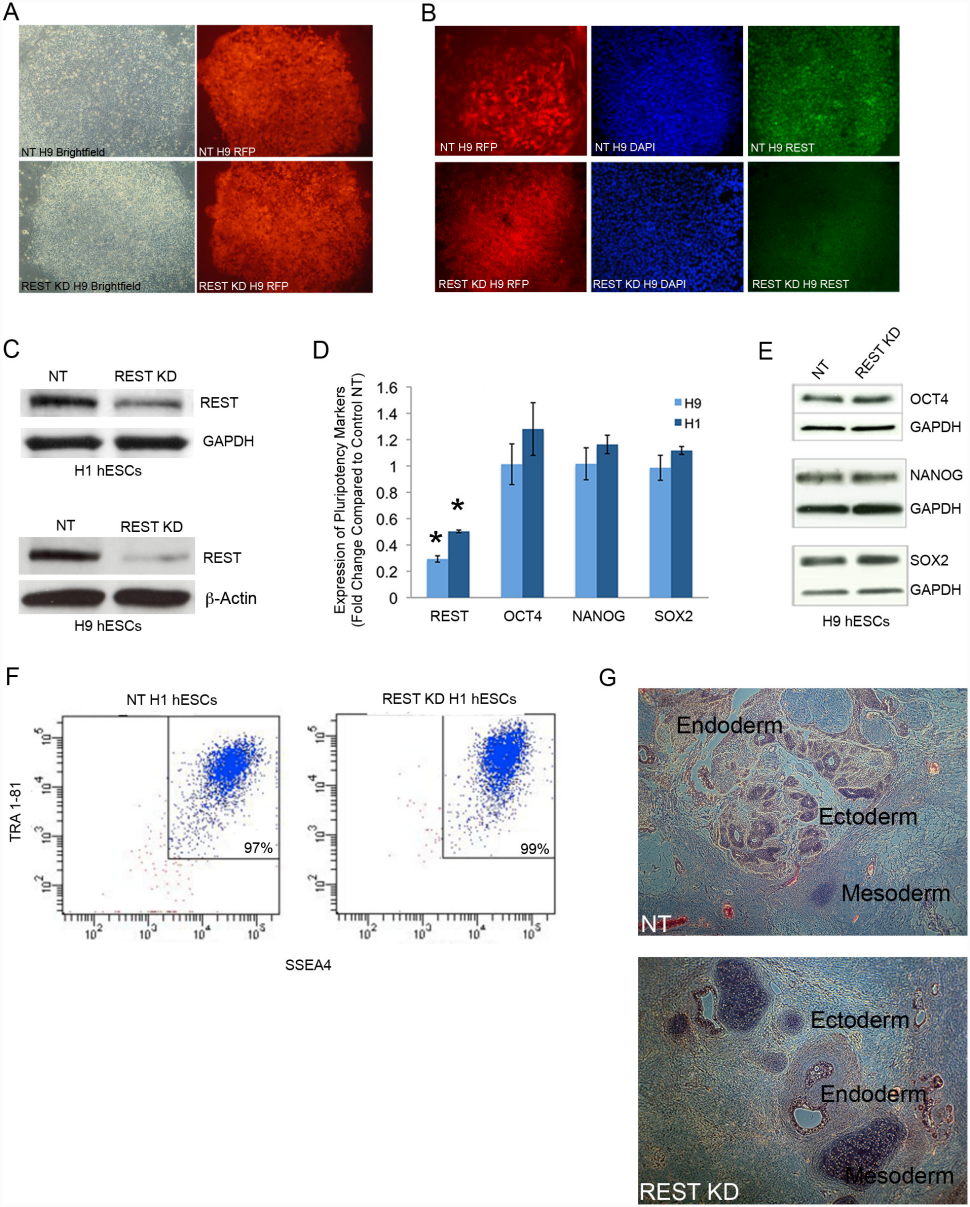
Knockdown (KD) of REST in human embryonic stem cells does not result in loss of pluripotency. **A**. H9 hESC colonies with an inducible shRNAmir expression system to knockdown REST (REST KD) or a scrambled shRNAmir Non-Target vector as a control (NT) are shown. RFP expression demonstrates near homogeneity of established NT or REST KD lines. **B**. Immunofluorescence analysis demonstrated reduced REST protein expression in REST KD H9 hESCs. **C**. Western Blot analysis demonstrated reduced REST protein expression in REST KD H9 and H1 hESCs. **D**. Expression of pluripotency markers using qPCR. REST levels are statistically significantly knocked down (p<0.0001) in H9 and H1 REST KD hESCs, and there is no change in the expression of OCT4, SOX2 or NANOG, compared to control NT lines. Error bars represent standard error of three independent experiments and asterisks denote a p value < .05 using Student’s t-test analysis. **E**. Western blot shows no change in protein levels of OCT4, SOX2 or NANOG in REST KD H9 hESCs compared to control NT. **F**. FACS analysis shows no change in SSEA4 and TRA1-81 levels in REST KD hESCs. **G**. REST KD and NT hESCs are pluripotent and form teratomas *in vivo*. Cells from all three germ layers are shown with H&E staining and labeling in black.

In order to evaluate the role of REST/NRSF in regulation of hESC fate, we utilized the inducible Tet-On TRIPZ vector (see Methods), in which doxycycline (DOX) activates the expression of a TurboRFP reporter in addition to the shRNAmir. REST shRNAmir vector was used to knockdown REST (REST KD), and a scrambled Non-Target shRNAmir vector was used as a control (NT). As shown in Fig 1A, we were able to develop largely homogeneous RFP positive colonies in both the control NT and REST KD hESC lines (H9 is shown). The presence of DOX was used to manually pick RFP positive cells and thus was required from the start for making each stable knockdown line. To verify REST KD, we evaluated protein (Fig 1B and 1C) and RNA (Fig 1D) levels, and found decreased REST expression in both cases. In addition, as REST is a transcriptional repressor, we verified that upon REST KD there is an increase in direct REST targets. Indeed REST targets including SYP, SYT4 and TRKC are increased upon REST KD (S1 Fig). In order to determine whether REST KD results in loss of expression of signature pluripotency markers, we performed qPCR (Fig 1D) and Western blot analysis (Fig 1E), and in both cases found no change in expression of pluripotency markers. To confirm that REST is not required for maintenance of hESCs, we performed FACS analysis for the signature pluripotency markers SSEA4 and TRA 1–81, and again did not see any decrease in expression compared to control NT hESCs (Fig 1F). We verified that REST KD did not result in a loss of pluripotency by using the gold standard *in vivo* teratoma assay, which demonstrated that both REST KD and control NT hESCs are able to generate teratomas with cells representing all three germ layers (Fig 1G).

**Fig 2:**
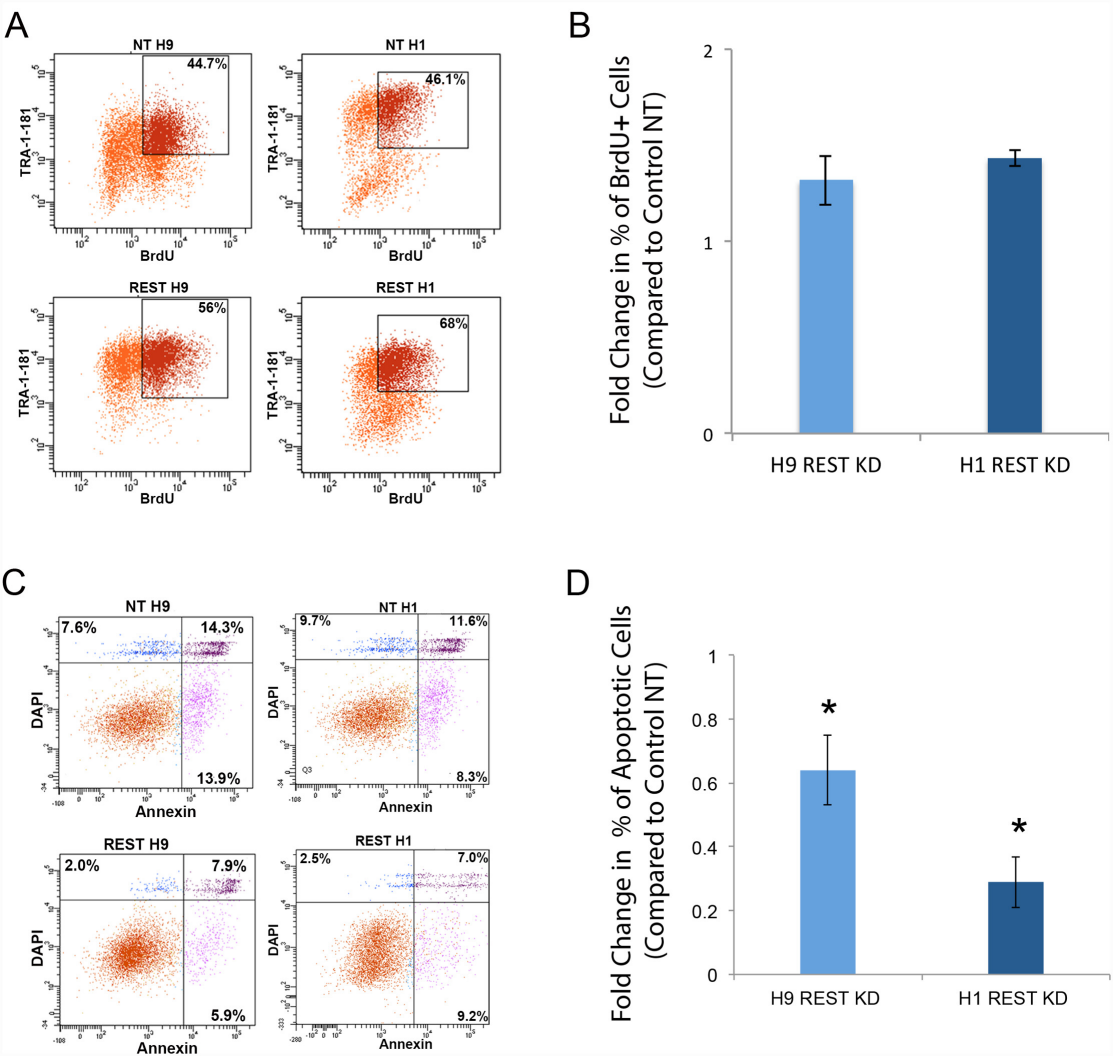
REST KD hESCs have increased survival. **A**. REST KD and NT hESCs (H1 and H9) were evaluated for BrdU incorporation by FACS analysis for TRA-1-81 and BrdU double positive cells. **B**. Percentage of TRA-1-81and BrdU positive cells were not statistically significantly different in REST KD compared to control NT cells. Error bars represent standard error of three independent experiments (performed at p52, p54 and p60 for H9 cells, and at p47, p49, and p50 for H1 cells). **C**. REST KD and NT hESCs (H1 and H9) were evaluated for the percentage of Annexin V and DAPI positive cells by FACS analysis. **D**. FACS analysis for Annexin V and DAPI staining demonstrated that REST KD hESCs have statistically significant improvements in survival as demonstrated by reduced levels of apoptotic cells (p<0.032 in H9 and p<0.002 in H1) compared to control NT hESCs. Error bars represent standard error of three independent experiments (performed at p55, p57 and p59 for H9 cells, and at p55, p56, and p57 for H1 cells) and asterisks denote a p value < .05 using Student’s t-test analysis.

Over long-term culture of the cells, we often observed that REST KD hESCs passaged/survived better than control NT lines (H9 REST KD cells were followed for 21 passages: p39 to p60, and H1 REST KD cells were followed for 33 passages: p38 to p71). To evaluate proliferation, we performed BrdU analysis, but did not find any significant differences in BrdU incorporation between control NT and REST KD H9 or H1 hESCs (Fig 2A and 2B). However, our observation of enhanced passaging/survival capacity in REST KD hESCs was validated by apoptosis analysis that indicated lower levels of apoptotic cells (i.e., cells positive for Annexin V and DAPI) among both H1 and H9 REST KD hESCs, compared to controls (Fig 2C and 2D). In summary, we found that REST is not required for hESC maintenance, but may play a role in regulation of hESC survival.

**Fig 3:**
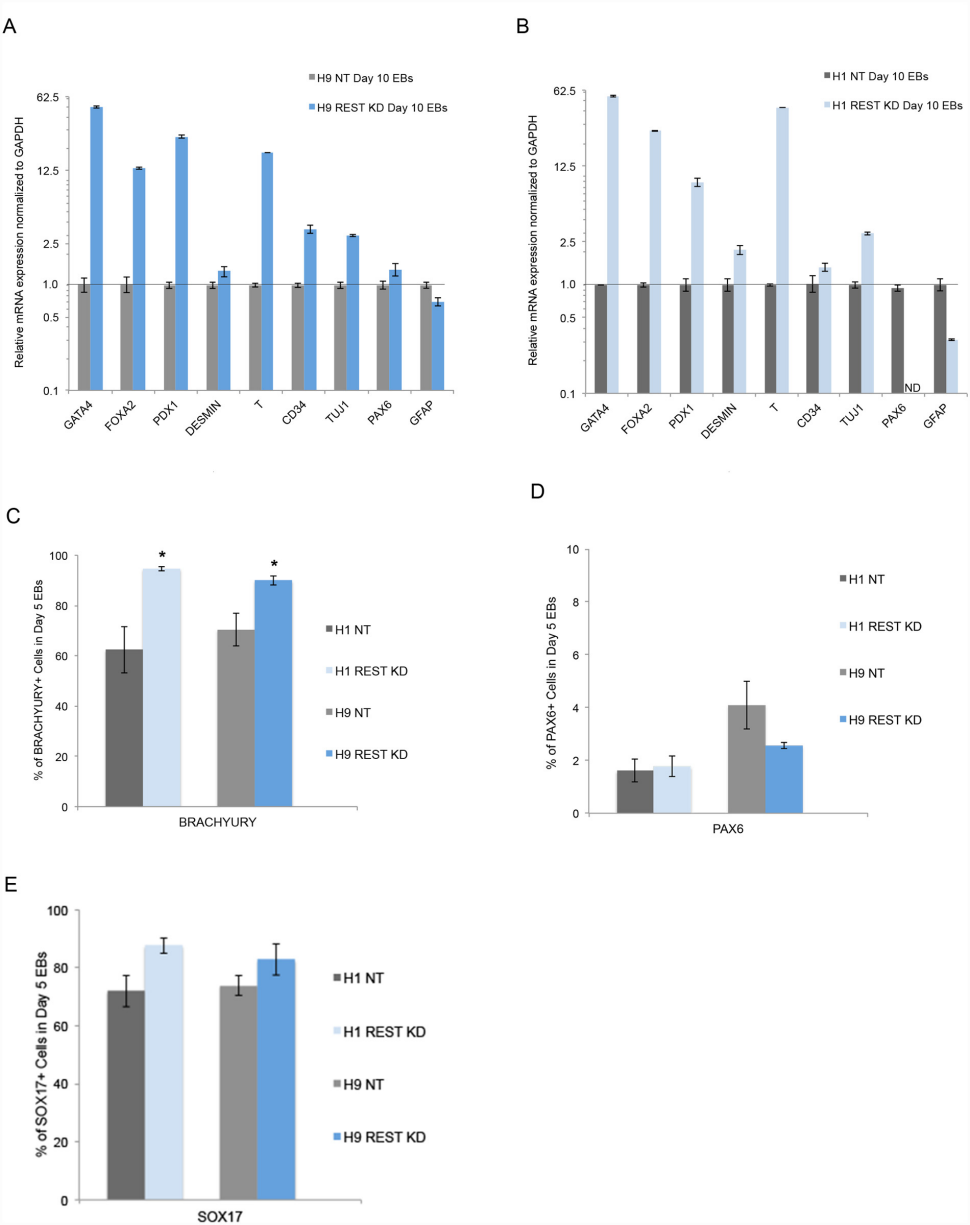
REST KD cells have increased mesendoderm lineage differentiation bias *in vitro*. **A-B**. Evaluation of *in vitro* differentiation potential in Day 10 embryoid bodies (EBs). QPCR analysis revealed an increase in expression of endoderm and/or mesoderm markers in both H9 (**A**.) and H1 (**B**.) REST KD day 10 EBs. Shown are representative graphs of lineage marker analysis for each of the three germ layers. Error bars represent standard error of the mean (SEM) from three technical replicates. ND = Not detected. **C-E**. Evaluation of protein changes using FACS analysis across two independent REST KD lines (H9, H1) revealed an increase in expression of the mesendoderm marker BRACHYURY compared to control NT lines but no change in the ectoderm marker PAX6 or endoderm marker SOX17. Error bars represent SEM of three independent experiments and asterisks denote a p value < .05 using Student’s t-test analysis.

In order to get a quantitative measure of the role of REST in differentiation, we performed *in vitro* analysis of the expression of at least two makers from each of the three germ layers across two independent hESC lines (H9, H1). We differentiated each hESC line under spontaneous embryoid body (EB) culture conditions and compared the expression of germ layer markers at Day 5 and Day 10 EB time points using qPCR (day 10 is shown). Interestingly, we found that REST KD hESCs had an increase in expression of endoderm or mesoderm markers compared to control NT hESCs across both cell lines (Fig 3A and 3B). To confirm the differentiation bias at the protein level, and to get a better understanding of the cell types present in REST KD and control NT EBs, we repeated the spontaneous EB differentiation, and used intracellular FACS analysis to evaluate three candidate lineage markers—SOX17, BRACHYURY and PAX6. The FACS analysis revealed that PAX6 and SOX17 were not statistically significantly different between REST KD and NT EBs (Fig 3D and 3E). However, REST KD EBs had a statistically significant increase in the percentage of BRACHYURY (mesendoderm) expressing cells across both cells lines (Fig 3C). Since the homeodomain protein MIXL1 plays a key role in mesendoderm specification [31], we analyzed MIXL1 expression via qPCR and immunohistochemistry. Day 5 and day 10 REST KD EBs showed increased MIXL1 mRNA expression compared to control NT EBs in the presence of doxycycline, but not in the absence of doxycycline (S2A and S2B Fig). Immunohistochemistry of day 5 EBs also revealed elevated MIXL1 protein expression and increased nuclear localization in REST KD EBs compared to control NT EBs (S2C Fig). In summary, although REST is not required for maintenance of hESC colonies, REST plays a role in regulating the early stages of EB differentiation.

In parallel to evaluating differentiation, we examined the role of REST KD with regard to genetic stability, using karyotype analysis (S1 Table). When NT and REST KD H1 p71 were sent out for G-band karyotype analysis, the NT cells were found to be normal, while the REST KD cells had trisomy 12. Analysis of NT H1 p50 and REST H1 p54 yielded similar results: all twenty NT cells were normal, while all twenty REST KD H1 cells had trisomy 12. For the H9 line, all twenty NT p55 cells were found to be normal, while all twenty REST KD p55 cells were found to be karyotypically abnormal. When we thawed earlier passage cells and sent REST KD H9 p44 for analysis, these too were found to have the same large pericentric inversion on chromosome 9 as the REST KD H9 p55 cells. We also used an siRNA mediated approach towards REST KD, where we performed repeated transfections approximately every 72hrs, and going up to 288hrs, of either scrambled siRNA or siRNA against REST. Copy number variant (CNV) analysis of NT and REST siRNA cells at 288hrs showed that both sets of cells were normal (S1 Table) demonstrating genomic stability. Thus, we found that REST KD resulted in karyotype instability in both H1 and H9 hESCs when targeted using shRNA but not when using siRNA. Importantly, the instability was not responsible for the differentiation bias seen, as no increase in endoderm or mesoderm markers was seen without addition of doxycycline, i.e., when the inducible promoter for the shRNA was not activated (S3A Fig). In order to further assess the effect of genetic instability on differentiation bias, we evaluated an independent hESC line that has previously been reported to have karyotypic abnormalities, including amplification of chromosomes 12 and 17 [32]. As shown in S3B–S3D Fig, BGO1V (V = variant or aneuploid) EBs did not have mesoderm or endoderm differentiation bias compared to control BGO1 EBs as measured by pPCR (S3B Fig) or FACS analysis (S3C and S3D Fig). This confirms previously published work demonstrating that aneuploidy does not predispose hESC lines to differentiate more efficiently or with a mesoderm/endoderm differentiation bias [33]. In fact, the two aneuploid lines examined in that study exhibited reduced endoderm differentiation.

**Fig 4:**
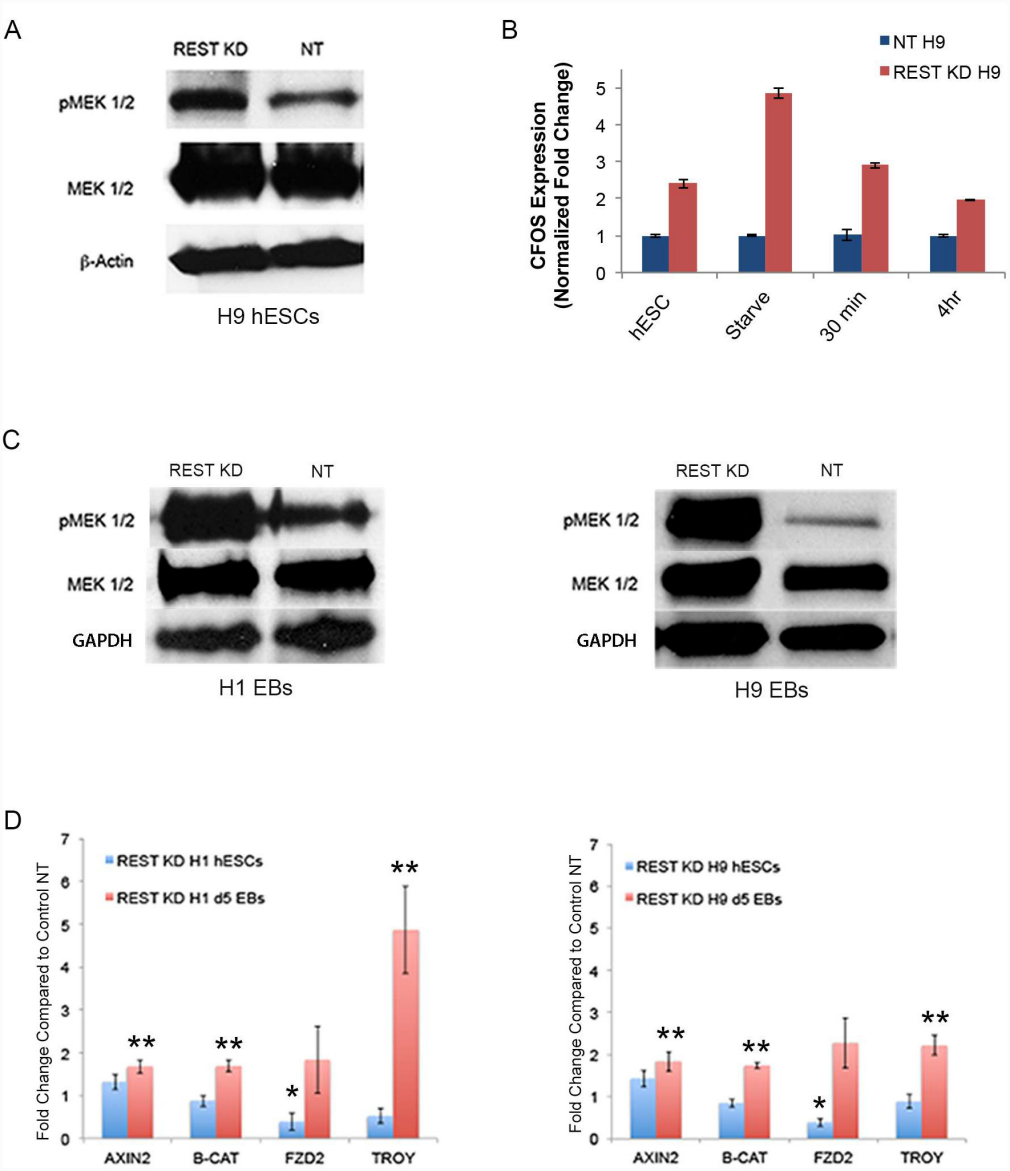
REST KD results in altered signaling including an increase in MEK and WNT activity in embryoid bodies (EBs). **A**. Western blot showing that REST KD H9 hESCs have increased pMEK1/2 (S217/221) expression compared to control NT H9 hESCs. MEK1/2 and β-ACTIN were used as loading controls. **B**. QPCR showing that REST KD H9 hESCs have increased expression of CFOS, a down-stream target of pMEK1/2. CFOS expression was examined in cells collected under four conditions: 24 hours after last feeding with media containing knockout serum (hESC); 24 hours after last feeding with media devoid of knockout serum (Starve); 30 minutes after switching from ‘Starve’ condition to knockout serum media; 4 hours after switching from ‘Starve’ condition to knockout serum media. REST KD hESCs had increased CFOS expression under all four conditions. **C**. Western Blot Analysis of Day 5 EBs for changes in MEK signaling revealed that pMEK 1/2 was increased in REST KD EBs. MEK ½ and GAPDH were used as loading controls. **D**. In order to evaluate WNT signaling, we evaluated expression of the WNT target genes AXIN2, Beta-CATENIN (B-CAT), FRIZZLED2 (FZD2) AND TROY in NT and REST KD hESCs as well as EBs, in H9 and H1 lines, via qPCR. Significant changes in expression of WNT target genes in REST KD hESCs are shown with a single asterisk (*). For both H1 and H9 REST KD hESCs, FZD2 was statistically significantly decreased (p<0.05). Significant changes in expression of WNT target genes in REST KD Day 5 EBs are shown with two asterisks (**). Across both lines of REST KD Day 5 EBs, we found a statistically significant increase in expression of WNT target genes AXIN2, B-CAT and TROY (p<0.022). Error bars represent SEM of three independent experiments and asterisks denote a p value < .05 using Student’s t-test analysis.

To determine if reduced REST levels in hESCs or EBs results in any change in pluripotency signaling, we evaluated four of the main pluripotency signaling networks (FGF/MEK, AKT, WNT and SMAD signaling). As shown in Fig 4A, we found elevated pMEK1/2 protein expression in REST KD hESCs compared to controls. CFOS, a key transcription factor downstream of the FGF/MEK/ERK pathway [32] also exhibited increased mRNA expression in REST KD hESCs compared to controls (Fig 4B). Elevated pMEK1/2 protein expression was also detected in REST KD EBs in both, H9 and H1 lines as compared to controls (Fig 4C). This trend was also seen in siRNA knockdown hESCs (S4 Fig) confirming that the changes seen in signaling are not a result of instability but are due to REST KD. Additionally, we saw a consistent increase in WNT signaling markers in REST KD EBs, across both, H1 and H9 lines (Fig 4D). Protein detection for SMAD and AKT signaling showed a slight increase in pSMAD 2/3 (S5A Fig), but no change in pAKT (S5B Fig) in REST KD hESCs. In summary, REST KD hESCs and EBs have altered pluripotency signaling, with REST KD EBs having increased MEK and WNT signaling during early lineage differentiation.

## Discussion

From our studies it is clear that reduced REST expression does not result in loss of pluripotency in human embryonic stem cells. REST KD hESCs express all of the traditional pluripotency markers and can differentiate into cells from all three germ layers. However, REST KD embryoid bodies (EBs) express higher levels of early mesendoderm markers as compared to control NT EBs. When REST was targeted using shRNA we found some form of genetic instability did occur in REST KD (i.e. deletions or amplifications), but not control NT cells. However, we did not see instability after REST KD using siRNA in short term or long term culture. This could be due to the fact that the targeting approaches were done with different KD platforms. Specific targeting of REST using genetic engineering approaches (ex. TALENS, ZINC Fingers or CRISPRs) will be required to clarify the role of REST in regulation of stability and differentiation across multiple targeting platforms. Importantly, the differentiation bias seen was not observed in the absence of doxycycline, i.e., when REST was not knocked down, confirming the role of REST in regulating lineage fate choice in hESCs.

REST KD hESCs were found to have a slight increase in pSMAD2/3, but no significant change in pAKT, even though they did demonstrate increased survival compared to control NT hESCs. Increased ERK/MEK/MAPK activity was detected in REST KD hESCs as well as REST KD Day 5 EBs. It has become clear that the balance of each of these signaling pathways is key to regulating the pluripotent state in hESCs [34]. Thus, the increase in pMEK 1/2 in REST KD hESCs is counterbalanced by no significant change in pAKT levels, which enables support of self-renewal in REST KD hESCs. Finally, we did see significantly increased WNT signaling in REST KD Day 5 EBs, but not in REST KD hESCs. Previous work has shown that the levels of WNT activation are critical to balancing lineage differentiation bias, with high levels of WNT signaling activating mesoderm and/or endoderm, and lower levels being sufficient to generate ectoderm [35]. Thus activation of MEK and WNT signaling in REST KD cells are likely to play key roles in the lineage bias seen in our EB studies.

Interestingly, Lu *et al*. [36] report that various neurotoxic stimuli in the ageing brains of healthy people cause increased expression of the REST protein, and this was partially dependent on the Wnt-signaling pathway. In this model, REST induction suppresses neuronal apoptotic cell death and the toxic effects of the accumulation of amyloid-β (Aβ, the main protein component of the plaques found in the brains of patients with Alzheimer's disease). In contrast in the brains of those with Alzheimer's disease, both Wnt signaling and REST induction are suppressed, leading to neurodegeneration. Although a different model system, it is clear that the role of REST and WNT signaling in regulation of cell survival/cell death will be important to evaluate to compare the role of REST regulation in survival and cell function across multiple cell lineages and disease models.

Critical hurdles for translation of the clinical potential of hPSCs into practice are their tumorigenic capacity, and the inefficiency in tailoring lineage differentiation. In this work, we have found that altering REST levels in hPSCs plays a role in regulating both pluripotency signaling and differentiation. Interestingly, activation of RAS/ERK/MAPK signaling, which is elevated in REST KD cells, has previously been shown to play a role in inhibiting neuroectoderm differentiation [37], as well as in regulating genetic stability in a number of systems [38, 39]. Importantly, it has recently been shown that members of the ERK and WNT superfamily are REST target genes in hESCs and differentiated cells [40], and provide mechanistic candidates for activation of the changes in cell growth and lineage differentiation seen in REST KD hESCs. Therefore future experiments will be directed towards understanding how REST loss regulates pluripotency signaling and improve our understanding of tailored lineage differentiation. Elucidating the role of REST in regulating cell fate of hPSCs could enable development of robust methods to stably culture and tailor lineage differentiation of these cells for use in regenerative medicine applications.

## Methods

### Ethics Statement

All work was performed according to approvals from the UCLA Institutional Animal Care and Use Committee (IACUC) #2006-119-23D and the UCLA Embryonic Stem Cell Research Oversight (ESCRO) Committee #2006-009-09C.

### Cell Culture and Development of Inducible REST KnockDown (KD) System in hESCs

hESCs used in this study were from lines H1 between 35–70 passages, and H9 between 35–70 passages. H1 and H9 cells were obtained from Wicell under MTA to UCLA Stem Cell Core Facility. hESCs were grown on gelatin-coated plates with mitomycin-C treated Mouse Embryonic Fibroblasts (MEFs) in hESC medium consisting of Dulbecco’s Modified Eagle Medium-F12 (DMEM/F-12, Invitrogen) supplemented with 20% Knockout serum (Invitrogen), 1mM non-essential amino acids, 1mM L-glutamine, 0.1 mM Beta-mercaptoethanol, 1% Penicillin/Streptomycin (Hyclone) and 4 ng/ml basic Fibroblast Growth Factor (Biological Resources Branch, National Cancer Institute). hESCs were routinely passaged every 5–7 days at a ratio of 1:2 or 1:3 depending on cell density. Briefly, hESCs were incubated with a sterile filtered collagenase IV (Invitrogen) solution (1mg collagenase/mL of DMEM/F-12) for five minutes at 37°C, physically dissociated into small clumps using a 5 ml pipette, collected in a conical tube, and centrifuged at 1,000 rpm for 5 minutes. The cell pellet was re-suspended in hESC media, and the cells were plated onto MEF plates. hESCs used for RNA or protein collection were grown on Matrigel (BD Biosciences) coated plates and fed MEF conditioned medium to reduce MEF contribution.

Non-target control hESCs (NT) and REST knockdown hESCs (REST KD) were generated using the TRIPZ vector (Open Biosystems, http://www.openbiosystems.com), a lentiviral inducible RNAi system with microRNA-adapted shRNA (shRNAmir). The TRIPZ vector contains a puromycin drug resistance marker, and a Tet-On system that induces expression of the shRNAmir and TurboRFP in the presence of doxycycline.

To make stable lines, hESCs were harvested with trypsin, and plated onto a matrigel coated 24 well plate at a density of 5 X 10^4^ cells/well using MEF conditioned media supplemented with 10μM of the ROCK inhibitor HA-1077 [41]. The day after cell plating, lentivirus containing non-target shRNA (NT) or REST shRNA (REST KD) was added to the cells at a final virus concentration of 3-5 X10^7^ TU/ml of MEF conditioned media. Polybrene was added to the media at a final concentration of 8ug/ml to aid transduction. The plates were spinoculated at 2,000 rpm at 37°C for four hours, then transferred to the incubator for overnight incubation. The next morning, the transduction cocktail was replaced with fresh conditioned media. 48hrs after transduction, and daily for 4 weeks thereafter, cells were fed conditioned media containing 1ug/ml puromycin for selection. 1ug/ml doxycycline was also added to the media to turn on expression of the shRNAmir and TurboRFP. RFP positive colonies were manually scraped to obtain a homogenous population of RFP expressing cells. NT and REST KD hESC cells were treated with collagenase and transferred onto feeders when ready for splitting.

### Embryoid Body (EB) Formation

Confluent hESCs colonies were detached from the feeder layer by incubating cells with 1 mg/ml collagenase for 30–60 minutes at 37°C. The detached colonies were washed off the plate, collected in a conical tube, and allowed to pellet. After aspirating the supernatant, the colonies were resuspended in EB medium (hESC medium without bFGF) and plated in ultra low attachment plates (Corning). EB media was replaced every other day.

### Teratomas

For teratoma formation, two confluent wells of hESCs were harvested as previously described [42]. At least 3 animals were injected for each cell line evaluated for teratomas. Cell pellets were resuspended in 50 μl of PBS and injected into the testis of 4–8 week old SCID beige mice (Charles River) according to UCLA-approved Animal Research Committee protocols. After 8 weeks, teratomas were isolated and fixed in 4% PFA for 24 hours. Fixed teratomas were embedded and processed by the Translational Pathology Core Laboratory at the David Geffen School of Medicine at UCLA. Teratoma sections were stained with H&E. All animal experiments were carried out according to IACUC approved methods. In brief for survival surgeries, all animals were anesthetized prior to testis injection and provided pain medication during and for 48 hours after surgeries. Euthanasia was carried out by UCLA approved IACUC procedures.

Additional methods can be found in S1 Methods.

## Supporting Information

S1 Fig REST target gene expression is increased upon REST KD in hESCs. H9 hESCs are shown but similar results were seen for H1 hESCs. REST levels are statistically significantly reduced in REST KD cells compared to control NT hESCs (p<0.0001). Direct REST target genes (SYP, SYT4 and TRKC) are statistically significantly increased in REST KD hESCs compared to control NT hESCs (p<0.004). Error bars represent SEM of three independent experiments and asterisks denote a p value < .05 using Student’s t-test analysis.
(PDF)

S2 Fig MIXL1 expression is increased in H9 REST KD EBs. **A**. QPCR analysis showing increased MIXL1 expression in doxycycline treated (+Dox) H9 REST KD day 5 EBs at all three passages tested: passage 49, 50 and 56. Increased MIXL1 expression was not seen in the absence of doxycycline (no Dox). Error bars represent SEM from three technical replicates. **B**. QPCR analysis for MIXL1 expression in H9 day 10 EBs. Again, MIXL1 was not increased under no Dox conditions, but was increased in +Dox REST KD EBs at all three passages tested: passage 48, 49 and 50. Error bars represent SEM from three technical replicates. **C**. Immunohistochemistry results demonstrating increased MIXL1 protein expression in H9 REST KD day 5 EBs (+Dox). **D**. To confirm REST is still knocked down during spontaneous EB formation (+ DOX), we evaluated REST levels by qPCR in REST KD compared to control NT EBs. As shown in this representative graph for H9 EBs, REST expression was decreased in day 5 and day 10 EBs. Error bars represent SEM from three technical replicates.
(PDF)

S3 Fig Mesoderm/ endoderm differentiation bias is not a consequence of aneuploidy. **A**. To confirm that the gene expression changes seen in EBs is a result of REST KD and not a consequence of aneuploidy, we evaluated expression of candidate markers from each of the three germ layers *in vitro* without addition of doxycycline (Dox). As shown in this representative graph for the H9 line, REST KD EBs did not have increased mesoderm/endoderm marker expression compared to controls under no Dox conditions, i.e., when the inducible promoter for the shRNA was not activated. Error bars represent standard error of the mean (SEM) from three technical replicates. **B**. Day 5 BGO1 and BGO1V EBs were evaluated for expression of candidate differentiation markers. QPCR analysis revealed that BG01V (aneuploid) EBs do not have elevated expression of endoderm/mesoderm markers compared to BG01 (control) EBs. Error bars represent standard error of the mean (SEM) from three technical replicates. **C**. FACS analysis of protein expression in Day 5 EBs demonstrates similar or reduced expression of SOX17, BRACHYURY or PAX6 in BGO1V compared to control BGO1. **D**. Quantitative representation of FACS analysis for lineage markers in BGO1 and BGO1V Day 5 EBs. Significant changes, calculated using an unpaired students t-test are shown with a single asterisk (*). Percentage of SOX17+ cells is significantly lower in the BGO1V line compared to BGO1 (p = 0.005). Percentage of BRACHYURY+ and PAX6+ cells is not significantly altered between the two lines.
(PDF)

S4 Fig CFOS expression is increased in UCLA1 REST siRNA KD hESCs. To confirm that elevated CFOS expression is not a result of aneuploidy in H9 REST KD cells, REST was transiently knocked down using siRNA in the UCLA1 line (REST KD UCLA1 siRNA) and compared to a scrambled non target control (NT UCLA1 siRNA). **A**. QPCR analysis showing decreased REST expression in REST KD UCLA1 siRNA cells. **B**. QPCR analysis showing increased CFOS expression in REST KD UCLA1 siRNA cells, demonstrating an increase in expression of a key transcription factor downstream of the FGF/ERK/MAPK pathway. Shown are representative graphs where error bars represent SEM from three technical replicates.
(PDF)

S5 Fig P-SMAD2/3 is increased and P-AKT signaling is not changed upon REST KD in hESCs. **A**. Western blot showing that REST KD H9 hESCs have increased pSMAD2/3 (S465/467) expression compared to control NT H9 hESCs. SMAD2/3 and β-ACTIN were used as loading controls. To evaluate the status of AKT signaling in REST KD hESCs we performed FACS analysis of TRA1-81, pAKT (Ser473) double positive hESCs. There was no statistically significant difference in percentage of TRA1-81, pAKT double positive REST KD hESCs compared to control NT hESCs.
(PDF)

S1 Methods (DOCX)

S1 Table Karyotypes from REST KD, Control NT and siRNA hESC lines. Genomic stability was evaluated using either G-band karyotype analysis or copy number variant (CNV) analysis. The CNV analysis for siRNA targeted cells was performed by the UCLA Clinical Microarray Core. The G-band karyotype analysis for shRNA targeted cells was performed by Cell Line Genetics, an independent provider of cell line characterization services. In all cases where a non-clonal aberration was observed in only one of the twenty cells analyzed, the karyotype was deemed a technical artifact by Cell Line Genetics. REST shRNA targeted lines were genetically unstable whereas REST siRNA KD and control siRNA lines were found to be genetically stable.
(DOCX)
