# Supplementary figures and images for: REST/NRSF Knockdown Alters Survival, Lineage Differentiation and Signaling in Human Embryonic Stem Cells
Source: PLoS One. 2015 Dec 21;10(12):e0145280. doi: 10.1371/journal.pone.0145280 (PMC4699193; doi:10.1371/journal.pone.0145280)

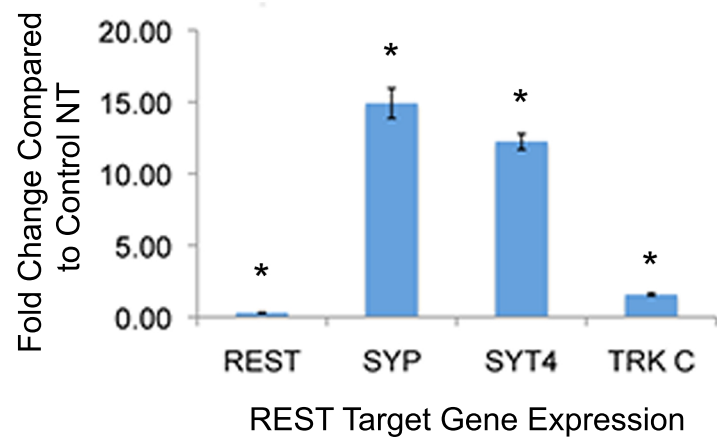

Supplement: S1 Fig — H9 hESCs are shown but similar results were seen for H1 hESCs. REST levels are statistically significantly reduced in REST KD cells compared to control NT hESCs (p<0.0001). Direct REST target genes (SYP, SYT4 and TRKC) are statistically significantly increased in REST KD hESCs compared to control NT hESCs (p<0.004). Error bars represent SEM of three independent experiments and asterisks denote a p value < .05 using Student’s t-test analysis. (PDF) [file pone.0145280.s001.pdf]

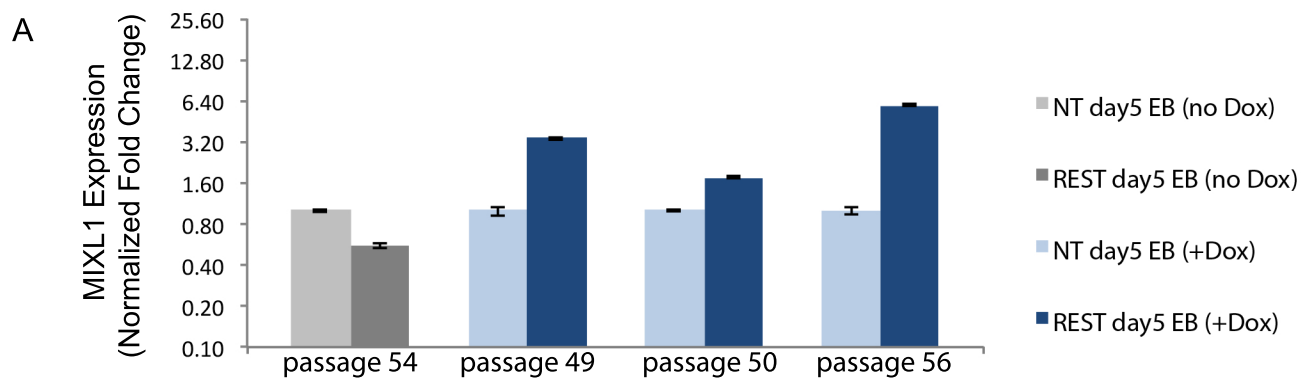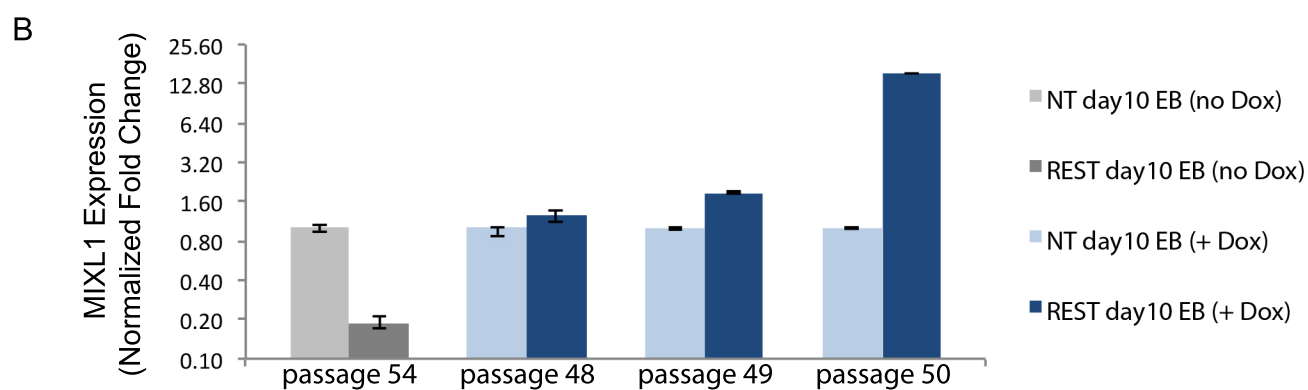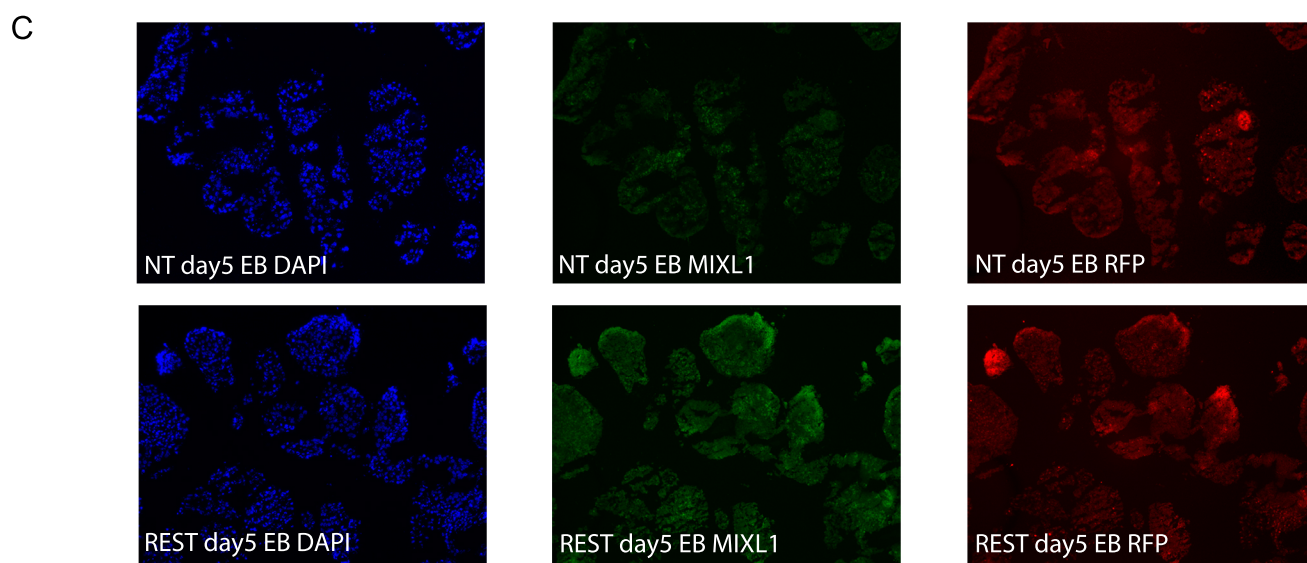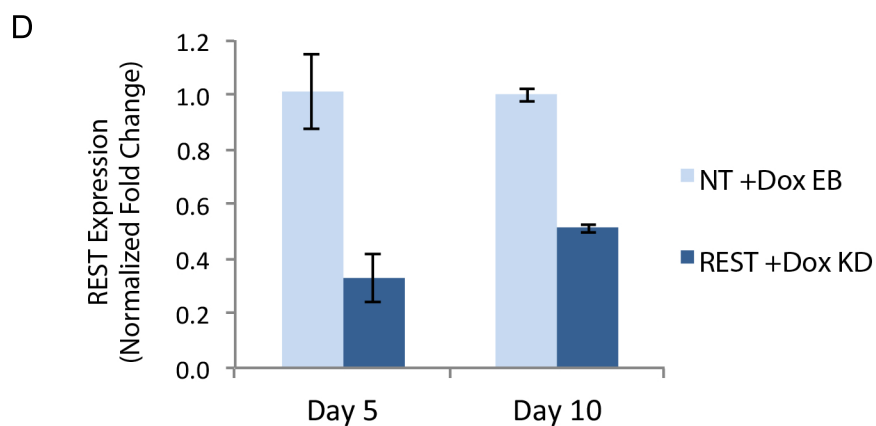

Supplement: S2 Fig — A. QPCR analysis showing increased MIXL1 expression in doxycycline treated (+Dox) H9 REST KD day 5 EBs at all three passages tested: passage 49, 50 and 56. Increased MIXL1 expression was not seen in the absence of doxycycline (no Dox). Error bars represent SEM from three technical replicates. B. QPCR analysis for MIXL1 expression in H9 day 10 EBs. Again, MIXL1 was not increased under no Dox conditions, but was increased in +Dox REST KD EBs at all three passages tested: passage 48, 49 and 50. Error bars represent SEM from three technical replicates. C. Immunohistochemistry results demonstrating increased MIXL1 protein expression in H9 REST KD day 5 EBs (+Dox). D. To confirm REST is still knocked down during spontaneous EB formation (+ DOX), we evaluated REST levels by qPCR in REST KD compared to control NT EBs. As shown in this representative graph for H9 EBs, REST expression was decreased in day 5 and day 10 EBs. Error bars represent SEM from three technical replicates. (PDF) [file pone.0145280.s002.pdf]

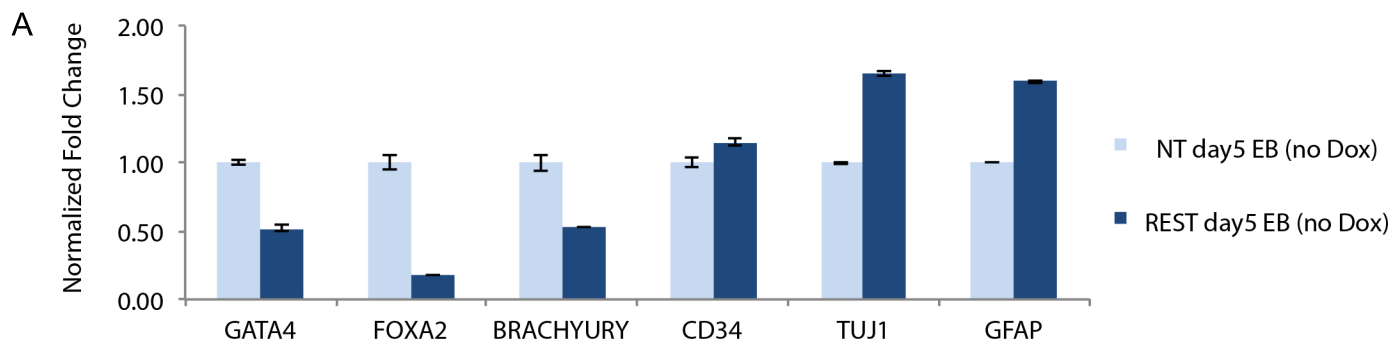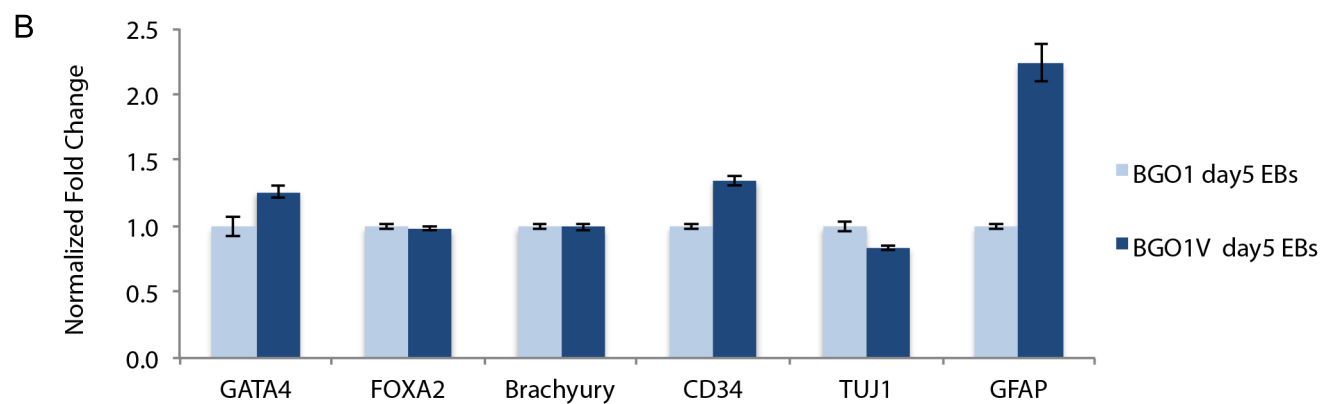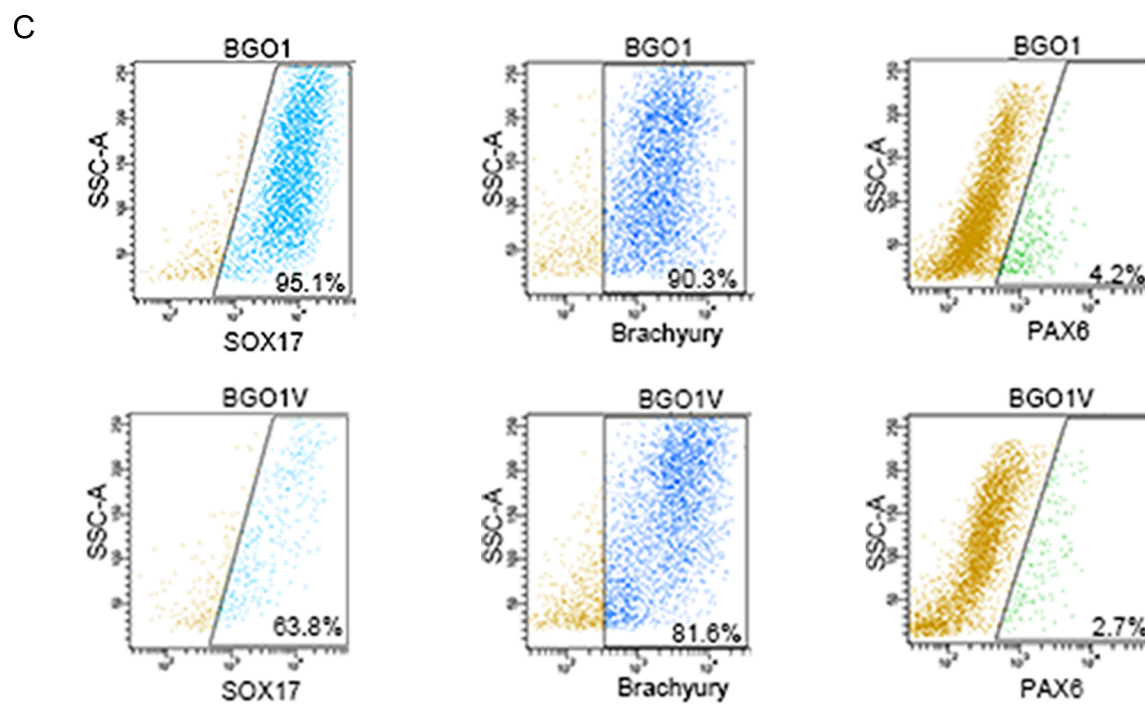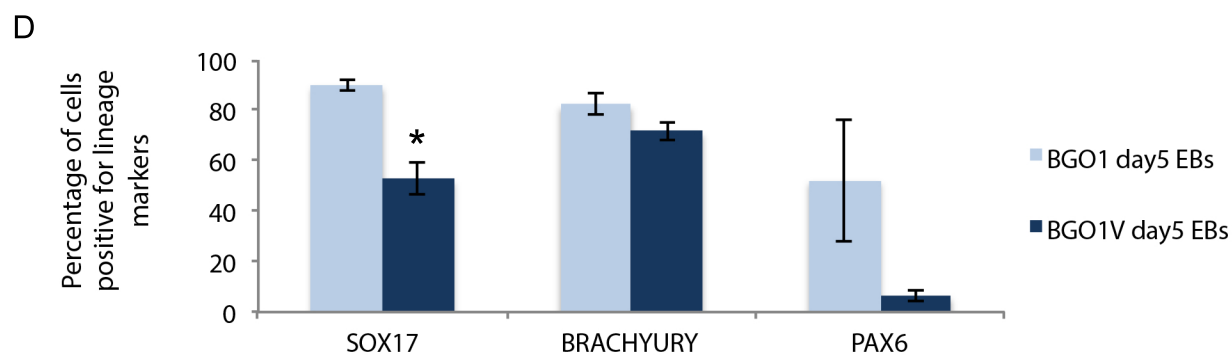

Supplement: S3 Fig — A. To confirm that the gene expression changes seen in EBs is a result of REST KD and not a consequence of aneuploidy, we evaluated expression of candidate markers from each of the three germ layers in vitro without addition of doxycycline (Dox). As shown in this representative graph for the H9 line, REST KD EBs did not have increased mesoderm/endoderm marker expression compared to controls under no Dox conditions, i.e., when the inducible promoter for the shRNA was not activated. Error bars represent standard error of the mean (SEM) from three technical replicates. B. Day 5 BGO1 and BGO1V EBs were evaluated for expression of candidate differentiation markers. QPCR analysis revealed that BG01V (aneuploid) EBs do not have elevated expression of endoderm/mesoderm markers compared to BG01 (control) EBs. Error bars represent standard error of the mean (SEM) from three technical replicates. C. FACS analysis of protein expression in Day 5 EBs demonstrates similar or reduced expression of SOX17, BRACHYURY or PAX6 in BGO1V compared to control BGO1. D. Quantitative representation of FACS analysis for lineage markers in BGO1 and BGO1V Day 5 EBs. Significant changes, calculated using an unpaired students t-test are shown with a single asterisk (*). Percentage of SOX17+ cells is significantly lower in the BGO1V line compared to BGO1 (p = 0.005). Percentage of BRACHYURY+ and PAX6+ cells is not significantly altered between the two lines. (PDF) [file pone.0145280.s003.pdf]

A

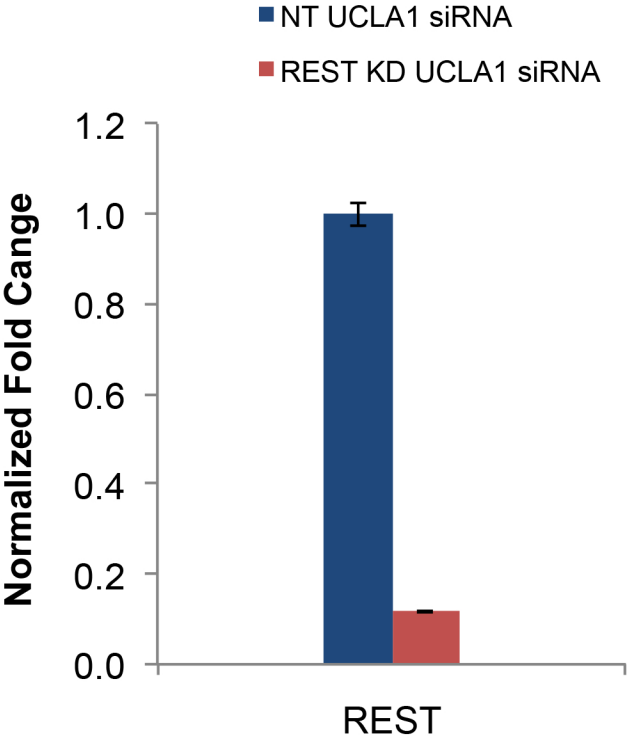

B

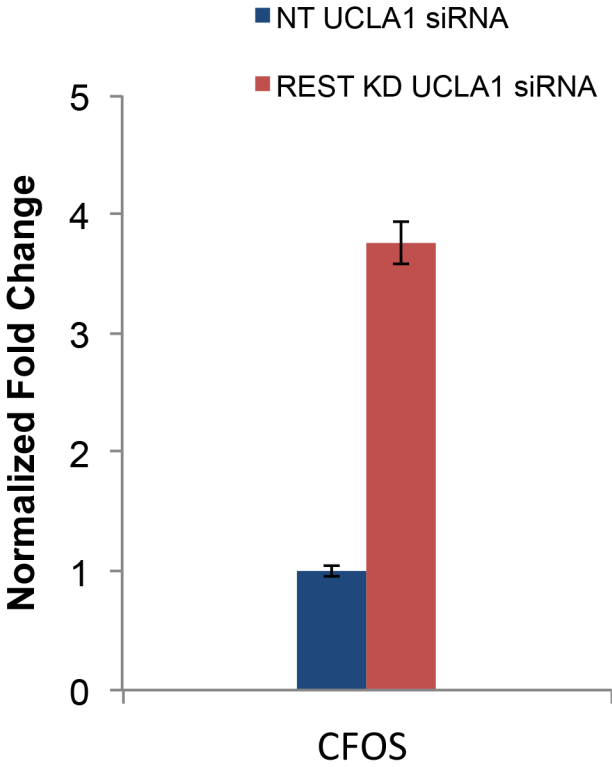

Supplement: S4 Fig — To confirm that elevated CFOS expression is not a result of aneuploidy in H9 REST KD cells, REST was transiently knocked down using siRNA in the UCLA1 line (REST KD UCLA1 siRNA) and compared to a scrambled non target control (NT UCLA1 siRNA). A. QPCR analysis showing decreased REST expression in REST KD UCLA1 siRNA cells. B. QPCR analysis showing increased CFOS expression in REST KD UCLA1 siRNA cells, demonstrating an increase in expression of a key transcription factor downstream of the FGF/ERK/MAPK pathway. Shown are representative graphs where error bars represent SEM from three technical replicates. (PDF) [file pone.0145280.s004.pdf]

A

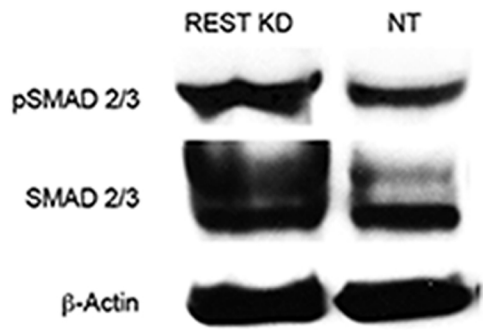

B

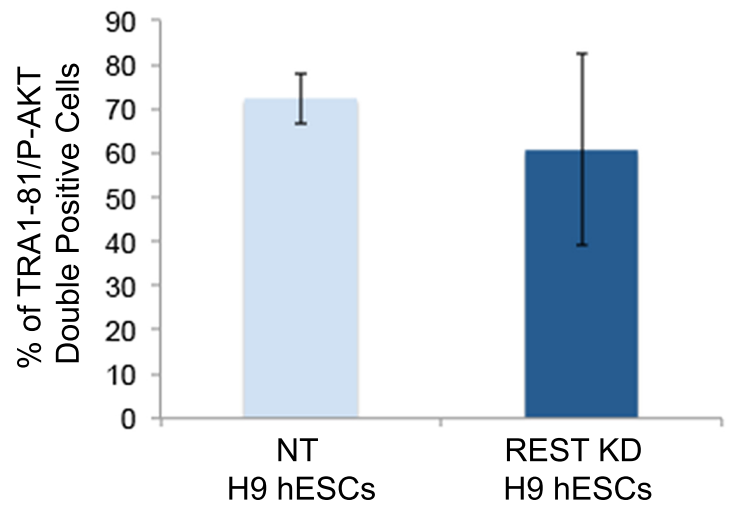

Supplement: S5 Fig — A. Western blot showing that REST KD H9 hESCs have increased pSMAD2/3 (S465/467) expression compared to control NT H9 hESCs. SMAD2/3 and β-ACTIN were used as loading controls. To evaluate the status of AKT signaling in REST KD hESCs we performed FACS analysis of TRA1-81, pAKT (Ser473) double positive hESCs. There was no statistically significant difference in percentage of TRA1-81, pAKT double positive REST KD hESCs compared to control NT hESCs. (PDF) [file pone.0145280.s005.pdf]
