# Supplementary material for: REST/NRSF Knockdown Alters Survival, Lineage Differentiation and Signaling in Human Embryonic Stem Cells
Source: PLoS One. 2015 Dec 21;10(12):e0145280. doi: 10.1371/journal.pone.0145280 (PMC4699193; doi:10.1371/journal.pone.0145280)
Supplement: S1 Methods — (DOCX) [file pone.0145280.s006.docx]

**S1 Methods**

**Cell death/ Cell Survival analysis**NT and REST KD hESC cells were trypsin harvested and plated at a density of 1X10^5^ cells/well of Matrigel coated 6 well plates, using MEF conditioned media supplemented with 10μM of the ROCK inhibitor HA-1077. Cells were cultured in conditioned media (without the ROCK inhibitor) for 5-7 days. Cell death was measured by staining with Annexin V (FITC Annexin V Apoptosis Detection Kit, BD Pharmingen) and DAPI followed by flow cytometry analysis.

**Karyotype Analysis**Karyotype analysis was performed on long-term cultured cells by Cell Line Genetics (Madison, Wisconsin http://www.clgenetics.com). Cells were prepared and shipped according to Cell Line Genetics procedures. Briefly, stable lines were generated as described in the “Cell Culture and Development of Inducible REST KnockDown (KD) System in hESCs” methods section of the paper. REST KD and Control NT hESCs were maintained in culture for at least 6 weeks prior to shipping for karyotype analysis. hESCs were passaged onto feeders in T‑25 flasks, and live cultures were sent for analysis of at least 20 metaphase spreads per sample. CNV arrays and analysis was performed by recharge using the UCLA Clinical Microarray Core (CMC).

**Western blot**Relative protein levels were detected using standard immunoblot techniques. The following primary antibodies were used: OCT4, NANOG, SOX2, pSMAD, SMAD, pMEK, and MEK (Cell Signaling), GAPDH (Abcam) and β-Actin (Santa Cruz Biotechnology). HRP-conjugated secondary antibodies (Promega) were detected by enhanced chemiluminescence (GE Lifesciences).

**Quantitative Real Time PCR (qPCR)**RNA was extracted using the RNeasy Mini Kit (Qiagen). cDNA was synthesized using the iScript cDNA synthesis kit (BioRad). RT-PCR was performed using FastStart Universal SYBR Green Master Mix (Roche). ViiA7 RT-PCR system was used for RT-PCR run (Applied Biosystems). Primer sequences are shown below.

| Gene | Forward primer | Reverse primer |
| --- | --- | --- |
| GAPDH | TGCCAAGGCTGTGGGCAAGGTCATCCCT | ACGGCAGGTCAGGTCCACCACTGACACG |
| REST | CGACATGCAAGACAGGTTCACAAT | AGCTGCATAGTCACATACAGGGCA |
| OCT4 | ACATCAAAGCTCTGCAGAAAGAACT | CTGAATACCTTCCCAAATAGAACCC |
| SOX2 | CTTTTGTTCGATCCCAACTTTC | ATACATGGATTCTCGGCAGAC |
| NANOG | CAGCTGTGTGTACTCAATGATAGATTT | ACACCATTGCTATTCTTCGGCCAGTTG |
| GATA4 | TACATCAGCTTCCGGAACCACCAA | ATCCAGCATTGAGCAAAGGGCTC |
| FOXA2 | GGAGCGGTGAAGATGGAA | TACGTGTTCATGCCGTTCAT |
| BRACHYURY | TGCTTCCCTGAGACCCAGTT | GATCACTTCTTTCCTTTGCATCAAG |
| CD34 | TGAAGCCTAGCCTGTCACCT | CGCACAGCTGGAGGTCTTAT |
| TUJ1 | GTACGAAGACGACCAGGAGG | GGGTTTAGACACTGCTGGCT |
| GFAP | GGGAGCTTGATTCTCAGCAC | CTGGGGTTAAGAAGCAGCAG |
| SYP | CTTTAAGCGAGGCAGAATGG | GCCTTGCTCAAGATCTGTCC |
| SYT4 | GGGCTGAGTTGAGAACACTGTGGC | ACTGAGGCTTGTGACAGGCAGTGA |
| TRKC | TGCAGTCCATCAACACTCACCAGA | TGTAGTGGGTGGGCTTGTTGAAGA |
| AXIN2 | ACAACAGCATTGTCTCCAAGCAGC | GCGCCTGGTCAAACATGATGGAAT |
| β-CATENIN | TGCAGTTCGCCTTCACTATGGACT | GATTTGCGGGACAAAGGGCAAGAT |
| FZD2 | GGAAGCATTCGCCTTTGAGCACTT | AACTGCTAACCTGAACGCCAGAGA |
| TROY | TGTCTAAGGAATGTGGCTTCGGCT | TCACTGGTGGCTGAACAATTTGCC |

**Immunocytochemistry**Slides were fixed with 4% PFA in PBS at room temperature for 15 minutes, washed thrice with PBS, and blocked for an hour in blocking buffer (0.3% Triton-X 100, 10% normal goat serum in PBS) prior to staining. Slides were incubated for 2hrs at room temperature with anti-REST (Millipore) primary antibody diluted 1:100 in antibody dilution buffer (0.1% Triton-X 100, 1% normal goat serum in PBS). After washing thrice with PBS, slides were incubated for 1hr in the dark at room temperature with Goat anti-rabbit FITC-conjugated secondary antibody (Pierce) diluted 1:500 in antibody dilution buffer. Slides were mounted in Vectashield Mounting medium for Fluorescence with DAPI (Vector Labs).

**Fluorescence-Activated Cell Sorting Analysis**For staining hESCs with an extracellular stem cell marker, cells were treated with trypsin to dissociate into single cells, rinsed with PBS, and blocked in FACS buffer (0.5% BSA in PBS) for 10 minutes at 4ºC. Cells were incubated with TRA-1-81 (1:100, Santa Cruz Biotechnology) primary antibody for 30 minutes at 4ºC, washed with FACS buffer, and then incubated with Cy5-conjugated goat anti-mouse secondary antibody (1:250, Jackson ImmunoResearch) for 20 minutes at 4ºC. To detect intracellular markers of differentiation, EBs were dissociated into single cells with trypsin treatment, rinsed with PBS, fixed with 2% PFA for 10 minutes at 37ºC and then permeabilized using 90% methanol for 30 minutes at 4ºC. After washing twice with FACS buffer and blocking for 10 minutes, cells were incubated with one of the following antibodies for 1hr at room temperature: the Alexa Fluor 488 conjugated mouse anti-human antibodies PAX6 or SOX17 (1:20 dilution, BD Biosciences), or APC-conjugated mouse monoclonal anti-human Brachyury (1:10 dilution, R&D Systems). FACS acquisition was performed using a LSR II FACS sorter and analyzed using FACS DIVA software (BD Biosciences).

**REST knockdown (KD) via serial siRNA transfection of UCLA1 hESCs**UCLA1 p19 hESC cells were trypsin harvested and plated at a density of 3.5X10^5^ cells/well of Matrigel coated 6 well plates, using MEF conditioned media supplemented with 10μM of the ROCK inhibitor HA-1077. After 24hrs, cells were transfected with 100nM of either scrambled non-target siRNA (NT), or REST siRNA (Dharmacon ON-TARGETplus SMARTpool siRNA). Cells were maintained in conditioned media, retransfected every three days, and passaged with trypsin when confluent. In total, the cells were transfected four times, and passaged 4 times, before being harvested and processed for CNV analysis at 288hrs (12 days, UCLA1 p23, data not shown).
