# Supplementary material for: REST/NRSF Knockdown Alters Survival, Lineage Differentiation and Signaling in Human Embryonic Stem Cells
Source: PLoS One. 2015 Dec 21;10(12):e0145280. doi: 10.1371/journal.pone.0145280 (PMC4699193; doi:10.1371/journal.pone.0145280)
Supplement: S1 Table — Genomic stability was evaluated using either G-band karyotype analysis or copy number variant (CNV) analysis. The CNV analysis for siRNA targeted cells was performed by the UCLA Clinical Microarray Core. The G-band karyotype analysis for shRNA targeted cells was performed by Cell Line Genetics, an independent provider of cell line characterization services. In all cases where a non-clonal aberration was observed in only one of the twenty cells analyzed, the karyotype was deemed a technical artifact by Cell Line Genetics. REST shRNA targeted lines were genetically unstable whereas REST siRNA KD and control siRNA lines were found to be genetically stable. (DOCX) [file pone.0145280.s007.docx]

| NT pTRIPZ H9 p55 | 46,XX[20] |
| --- | --- |
| REST pTRIPZ H9 p55 | 46,XX,inv(9)(p22;q22.3)[19]  46,XX,inv(9)(p22;q22.3),t(13;22)(q32;q13)[1; most likely a technical artifact] |
| REST pTRIPZ H9 p44 | 46,XX,inv(9)(p22;q22.3)[20] |
| NT pTRIPZ H1 p50 | 46,XY[20] |
| REST pTRIPZ H1 p54 | 47,XY,+12[19]  45,Y,+12,-20[1; most likely a technical artifact] |
| NT pTRIPZ H1 p71 | 46,XY[18]  47,XY,+3[1; most likely a technical artifact]  45,XY,-12[1; most likely a technical artifact] |
| REST pTRIPZ H1 p71 | 47,XY,+12[19]  46,XY,-8,+12[1; most likely a technical artifact] |
| NT siRNA 288hr UCLA1 p23 | 46,XX (CNV Analysis) |
| REST siRNA 288hr UCLA1 p23 | 46,XX (CNV Analysis) |

**S1 Table. Karyotypes from shRNA and siRNA mediated REST KD and Control NT hESC lines.**
